# Prospects of Hydrogels in Wound Healing: Toward the Next Generation of Smart Biomaterials

**DOI:** 10.3390/pharmaceutics17111486

**Published:** 2025-11-18

**Authors:** Cristina Casadidio, Roberta Censi

**Affiliations:** ChIP Chemistry Interdisciplinary Project Research Centre, School of Pharmacy, University of Camerino, Via Madonna delle Carceri, 62032 Camerino, MC, Italy; roberta.censi@unicam.it

## 1. Introduction

Wound healing continues to represent a major clinical challenge worldwide, particularly in the context of chronic wounds, burns, and infection-prone injuries that are increasingly complicated by antimicrobial resistance. Traditional dressings often act as passive barriers, providing protection but limited therapeutic benefit. In contrast, hydrogels have emerged as highly versatile biomaterials with the potential to transform wound management [1,2,3]. Their high water content, biocompatibility, and structural similarity to the extracellular matrix create a moist environment conducive to tissue repair, while their tunable networks allow for the incorporation and controlled release of therapeutic agents such as antimicrobials, growth factors, and anti-inflammatory compounds [4,5,6]. Recent years have seen a rapid expansion in hydrogel research, with PubMed entries on ‘hydrogels wound healing’ increasing more than fourfold since 2020 and remaining high through 2025. This surge reflects a shift toward multifunctional and stimulus-responsive designs capable of addressing infection, modulating inflammation, and adapting dynamically to different phases of the healing process [7,8,9]. Innovations include pH-buffering and antibacterial systems, natural product-infused composites, and hybrid formulations incorporating nanoparticles, each contributing to the evolution of hydrogels from passive dressings into intelligent, bioactive platforms [10,11,12,13]. Despite these advances, translation from preclinical promise to clinical practice remains limited. Challenges include variability in long-term efficacy, mechanical stability across wound types, the scalability of production, and regulatory hurdles. Bridging these gaps requires a focus on patient-centered design, the standardization of evaluation protocols, and the integration of hydrogels into broader regenerative strategies. Against this backdrop, this Special Issue, “Prospects of Hydrogels in Wound Healing”, highlights state-of-the-art developments and emerging research directions. By emphasizing multifunctionality, adaptability, and clinical relevance, this Special Issue underscores how engineered hydrogels are poised to shape the future of wound care, advancing beyond simple coverage toward active, smart therapies that optimize healing outcomes.

## 2. Overview of the Published Works

The first advances in this field developed from research into physically cross-linked PVA cryogels, where formulation composition and freezing conditions were shown to strongly influence swelling, porosity, and mechanical performance [Contribution 1]. These systems avoid toxic cross-linkers, offer high elasticity and durability, and, when optimized under −80 °C freezing, form interconnected pores that support comfort and resilience. Their flexibility means that they are particularly well suited for tailoring dressings to wounds with differing exudate levels. Building on structural tunability, another study explored the integration of acid-buffering properties into alginate gels to actively shape the wound microenvironment [Contribution 2]. By stabilizing the pH at 4.5, these gels suppressed biofilms of resistant bacteria while still supporting cell migration and wound closure. The balance between antimicrobial efficacy and tissue compatibility, best achieved in the ABF-3 formulation, highlights the potential of these gels as topical tools for infection control and as antibiotic-sparing alternatives in chronic wound management. Complementary to these synthetic systems, natural product-based formulations have gained attention. Plant-enriched hydrogels and oleogels were tested in a rat burn model, with oleogel preparations containing Boswellia and basil extracts showing superior outcomes, accelerating contraction, reducing inflammation, and enhancing epithelialization compared with hydrogel counterparts [Contribution 3]. Extending such strategies into veterinary medicine, equine trials with Bacuri butter and onion-based films offer insights into culturally rooted, biodegradable wound dressings [Contribution 4]. While these natural treatments did not significantly outperform standard care, this work demonstrates the relevance of horses as translational models and the possibility of cost-effective interventions in underexplored clinical contexts. Finally, hybrid designs merging bioactive nanomaterials with polymer networks exemplify the frontier of multifunctional dressings. Hydrogels infused with biogenic silver nanoparticles and oregano essential oil provided broad-spectrum antimicrobial activity against multidrug-resistant strains in burn models, while maintaining biocompatibility and stability [Contribution 5]. This synergy of green nanotechnology and natural compounds represents a sustainable approach to infection-prone wounds. Taken together, these studies underscore the versatility of hydrogel- and oleogel-based wound therapies. From finely tuned cryogel structures to acid-buffering systems, plant-derived extracts, and silver nanoparticle composites, current innovations demonstrate how wound dressings are evolving beyond passive barriers toward multifunctional platforms for infection control, tissue regeneration, and adaptable patient- or species-specific care.

Beyond skin and soft tissue repair, hydrogel systems are increasingly being applied in hard tissue regeneration. In peri-implant contexts, where healing is often compromised by bone loss or systemic conditions, hydrogels provide a biocompatible scaffold that mimics the extracellular matrix and enables the controlled delivery of osteogenic factors [Contribution 6]. Functionalization with agents such as BMP-2 or hydroxyapatite nanoparticles has shown promise in promoting osseointegration, angiogenesis, and mechanical stability around implants. While these advances remain largely preclinical, they illustrate how the same design principles, structural tunability, bioactivity, and multifunctionality that drive progress in wound dressings are also shaping next-generation strategies for bone and dental regeneration.

## 3. Advancing Hydrogel Strategies for Wound Healing

Wound healing remains a major global health challenge, particularly in aging populations, low-resource settings, and among patients suffering from chronic wounds, burns, and diabetic ulcers. Traditional dressings provide only passive protection and often fail in complex biological environments where infection, inflammation, and impaired vascularization coexist. This leads to a prolonged recovery time, higher healthcare costs, and reduced quality of life. Hydrogels, with their capacity to maintain hydration, deliver bioactives, and adapt structurally, offer a transformative opportunity to address these shortcomings. However, the path from laboratory innovation to accessible, scalable clinical solutions remains complicated by obstacles, including regulatory approval, sustainable production, and equitable distribution. To enable real-world impacts, future hydrogel development must be guided not only by scientific excellence but also by translational feasibility and sustainability. Emerging directions in hydrogel research reflect a shift toward more intelligent, multifunctional, and patient-centered solutions. A central strategy is the development of smart, stimulus-responsive hydrogels that can sense wound-specific stimuli such as pH changes, enzymatic activity, reactive oxygen species, or electrical fields. These systems could release antimicrobials, anti-inflammatory agents, or regenerative factors in real time, enabling dynamic, on-demand therapy and reducing the risk of infection or delayed healing. Beyond single triggers, sequential and multiphase delivery systems represent another crucial approach, enabling hydrogels to match the staged nature of wound healing by first promoting hemostasis and angiogenesis, then mitigating inflammation, and finally supporting tissue regeneration. The integration of nanotechnology, 3D bioprinting, and bioelectronics is expected to significantly expand hydrogel functionality. Nanoparticle incorporation may enhance antimicrobial, antioxidant, or gene-modulating capabilities, while 3D bioprinting enables the creation of customized scaffolds tailored to patient-specific wound geometries. Bioelectronic integration could allow the real-time monitoring of wound healing progression through biosensors embedded directly within the material. These approaches are particularly promising for burn victims and chronic ulcer patients, where conventional dressings cannot adapt to irregular wound beds.

Equally important are strategies for clinical translation and scalability. While preclinical studies consistently demonstrate promise, large, multicenter randomized clinical trials are urgently needed to establish long-term safety, efficacy, and cost-effectiveness. Success in this domain requires coordinated advances in GMP manufacturing, reproducible synthesis protocols, and regulatory science. Parallel to this, the expansion of hydrogel applications into veterinary medicine and low-resource settings highlights the importance of cost-effective, culturally acceptable, and locally sourced formulations.

**Figure 1:**
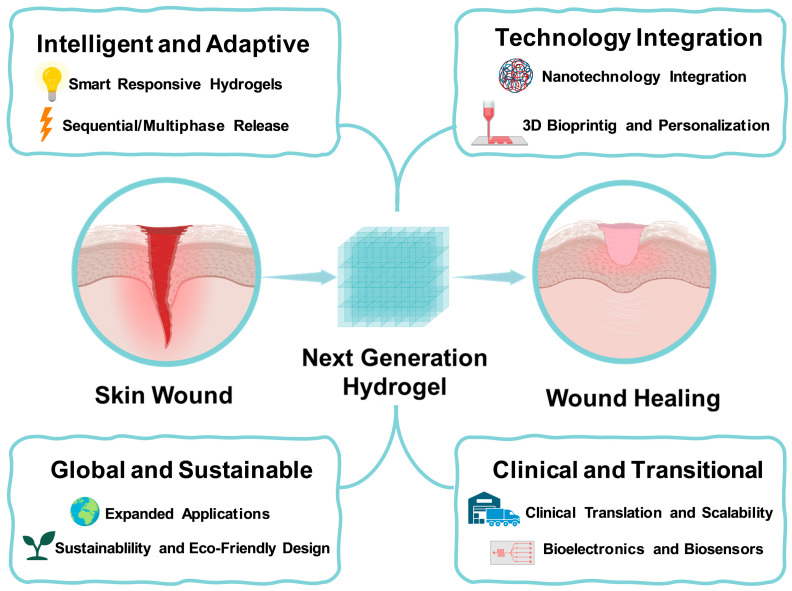
Scheme summarizing future perspectives in hydrogel-based wound healing (this scheme was partially created in Biorender, https://BioRender.com/4p2hkiw, (accessed on 9 November 2025)).

Furthermore, the next generation of hydrogels must align with the principles of sustainability and global health equity. This involves sourcing biodegradable, non-toxic polymers, employing green chemistry for nanoparticle synthesis, and minimizing ecological impacts throughout the production cycle. By embedding sustainability into biomaterial design, researchers and industry stakeholders can ensure that hydrogel technologies are not only clinically effective but also environmentally responsible and globally accessible. Together, these strategies mark a paradigm shift: hydrogels are moving beyond passive wound covers toward active, intelligent therapeutic systems that combine precision medicine with scalability and sustainability (Figure 1). If these innovations are successfully translated, they hold the potential to revolutionize wound care, reduce healthcare disparities, and improve patient outcomes worldwide.

## 4. Conclusions

Hydrogels represent a next-generation wound management strategy, evolving from passive protective dressings into dynamic, bioactive platforms capable of addressing the multifaceted challenges of tissue repair. The articles presented in this Special Issue highlight some possibilities of current innovations, ranging from structurally tunable cryogels and acid-buffering alginate systems to natural product-infused composites and hybrid nanomaterial designs. Together, they showcase how multifunctionality, adaptability, and sustainability are becoming defining features of hydrogel development. Looking forward, the translation of these advances into clinical practice will depend on rigorous long-term trials, scalable and eco-conscious manufacturing, and equitable distribution strategies that extend benefits to diverse patient populations, including those in low-resource settings. By combining scientific innovation with clinical and societal priorities, hydrogels have the potential not only to accelerate wound healing but also to transform standards of care across human and veterinary medicine, ultimately bridging the gap between laboratory discovery and global healthcare impacts.
